# The role of vitamin D receptor signaling in hair follicle health and alopecia: Current understanding and therapeutic implications

**DOI:** 10.1002/ccs3.70060

**Published:** 2025-12-29

**Authors:** Liancheng Guan, Fan Yang, Meijuan Li, Yujia Chen, Zexin Zhao, Hongxia Li, Deping Luo, Qian Li, Yunzhi Chen

**Affiliations:** ^1^ The Second Affiliated Hospital of Guizhou University of Traditional Chinese Medicine Guiyang Guizhou China; ^2^ Guizhou University of Traditional Chinese Medicine Guiyang Guizhou China

**Keywords:** alopecia, drug delivery, gene regulation, hair follicle, personalized medicine, vitamin D receptor

## Abstract

Vitamin D receptor (VDR) signaling plays a crucial role in hair follicle biology and represents a promising therapeutic target for various forms of alopecia. This review comprehensively examines the molecular mechanisms of VDR signaling in hair follicle development, cycling, and pathology. We discuss key molecular mechanisms of VDR‐dependent gene regulation through chromatin remodeling, transcriptional regulation, and recruitment of coregulatory complexes, which collectively regulate hair follicle homeostasis. Recent advances in understanding VDR genetic polymorphisms and their impact on treatment responses have provided new insights into personalized therapeutic approaches. The review explores current therapeutic strategies, from conventional vitamin D supplementation to emerging targeted delivery systems and combination therapies. We also analyze the challenges and limitations in current research, including the need for improved delivery systems and reliable biomarkers for treatment response prediction. The integration of molecular insights with clinical applications suggests promising directions for developing more effective, personalized treatments for various forms of alopecia. This comprehensive analysis underscores the significance of VDR‐targeted approaches in the future management of hair disorders and highlights the importance of continued research in this field.

## Graphical abstract

The vitamin D receptor (VDR) regulates hair follicle biology through ligand‐dependent and ligand‐independent mechanisms by heterodimerizing with retinoid X receptor, recruiting coactivator complexes, and activating target genes including Wnt inhibitors, hair keratins, and cell cycle regulators. VDR integrates with Wnt/β‐catenin, hedgehog, Notch, and BMP pathways to orchestrate hair cycling by promoting anagen initiation, regulating catagen transition, and maintaining stem cell quiescence. VDR dysfunction causes alopecia in knockout models and hereditary vitamin D‐resistant rickets patients, while VDR polymorphisms and reduced expression correlate with treatment variability in alopecia areata and androgenetic alopecia, and vitamin D deficiency associates with telogen effluvium. Current therapies include oral vitamin D supplementation, topical calcipotriol, and combination treatments with minoxidil, showing improved efficacy in female pattern hair loss and autoimmune alopecia. Emerging strategies encompass novel VDR agonists, advanced nanocarrier systems for enhanced follicular delivery, and gene therapy for VDR variant patients. Personalized approaches integrating VDR polymorphism profiling with biomarker‐guided treatment selection enable more effective, individualized therapies for alopecia based on comprehensive understanding of VDR's roles in hair follicle homeostasis.

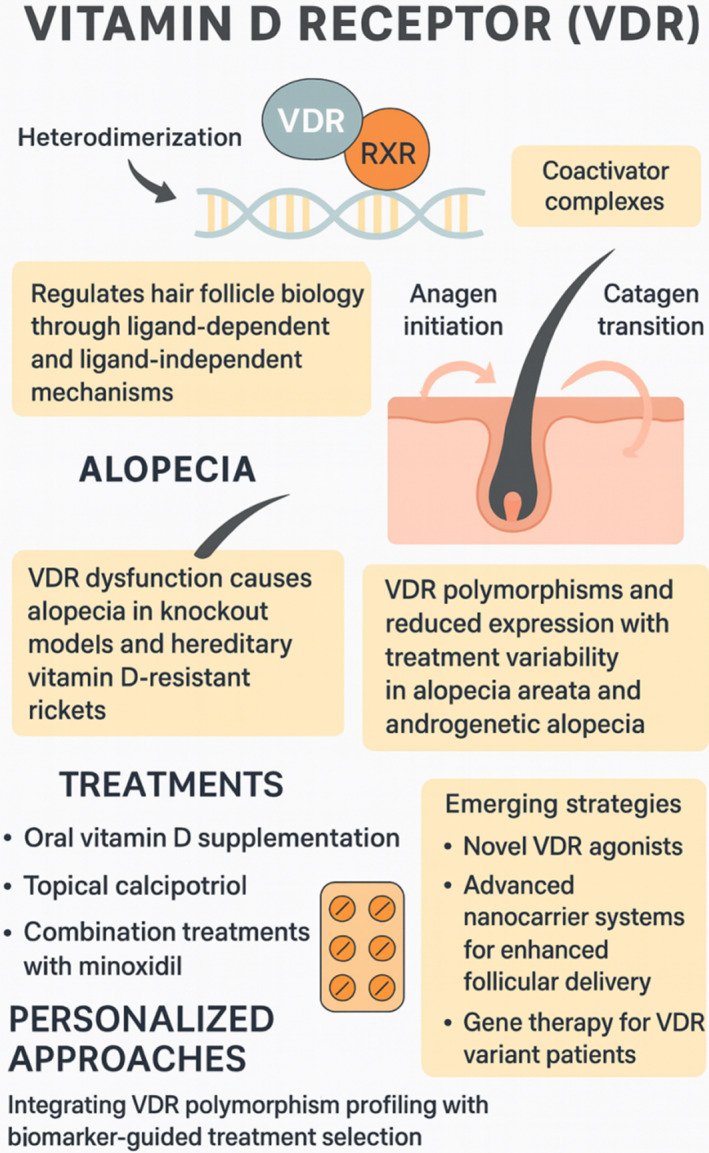

## 1 INTRODUCTION

Hair follicles represent one of the most complex mini‐organs in mammals, characterized by their unique ability to undergo continuous cycling throughout life.^1^, ^2^ This cycling process consists of three main phases: anagen (growth), catagen (regression), and telogen (resting), each regulated by precise molecular signals and cellular interactions.^3^, ^4^ The hair follicle structure comprises multiple cellular components, including the dermal papilla, matrix cells, and various epithelial layers, organized in a highly sophisticated arrangement.^5^, ^6^ These components interact through complex signaling networks involving Wnt/β‐catenin, Sonic hedgehog, Notch, and bone morphogenetic protein (BMP) pathways, which orchestrate the cycling process and maintain follicular homeostasis.^7^, ^8^

Hair loss disorders represent a significant global health concern, affecting millions of individuals worldwide and substantially impacting their quality of life.^9^ Androgenetic alopecia (AGA), the most prevalent form, affects up to 80% of men and 50% of women during their lifetime.^10^, ^11^ Other common conditions include alopecia areata (AA), an autoimmune disorder that affects approximately 2% of the population, and telogen effluvium, which is often triggered by various physiological stressors.^9^, ^12^ These conditions not only produce physical symptoms but also result in considerable psychological distress, including decreased self‐esteem, anxiety, and depression.^13^, ^14^ Despite the availability of treatments, many patients remain inadequately managed, underscoring the need for new therapeutic approaches.^15^

The vitamin D receptor (VDR) signaling system has emerged as a crucial regulator of hair follicle biology and potential therapeutic target for hair disorders.^16^ VDR, a member of the nuclear receptor superfamily, functions as a ligand‐dependent transcription factor, responding to vitamin D and its metabolites.^17^ The critical role of VDR in hair biology was first established through observations of alopecia in both VDR knockout mice and humans with hereditary vitamin D‐resistant rickets (HVDRR).^18^, ^19^ Subsequent research has revealed that VDR functions in hair follicles extend beyond its classical role in vitamin D signaling, including ligand‐independent actions and interactions with other signaling pathways.^20^, ^21^ Understanding these mechanisms has opened new avenues for therapeutic intervention in various hair disorders.

The complex relationship between VDR signaling and hair follicle biology represents a crucial frontier in dermatological research and therapeutic development. Our understanding has evolved from basic molecular mechanisms of VDR structure and function to sophisticated insights into its diverse roles in hair follicle cycling and pathological conditions. In this review, we summarize the fundamental mechanisms of VDR signaling in hair follicle biology, including its regulation of key downstream targets and chromatin remodeling. We delineate its critical roles in hair cycle phases and stem cell maintenance through interactions with various signaling pathways. We also discuss recent developments in therapeutic approaches targeting the VDR pathway, from conventional vitamin D supplementation to emerging gene therapy approaches and advanced nanocarrier‐based delivery systems. Finally, we explore current challenges and future directions in translating these molecular insights into effective clinical interventions for various forms of alopecia.

## 2 MOLECULAR BASIS OF VDR SIGNALING IN HAIR BIOLOGY

### 2.1 VDR structure and functions in skin and hair biology

**FIGURE 1:**
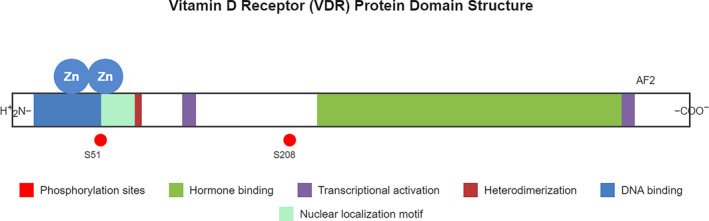
Structural organization of the human VDR protein. The schematic illustrates the functional domains and motifs of the human VDR across its amino acid sequence. The N‐terminal (H_2_N^+^) region contains two zinc finger motifs (Zn) that mediate DNA binding. The central region includes motifs involved in nuclear localization and heterodimerization with RXR. The C‐terminal portion encompasses the ligand (hormone) binding domain, which also contains the AF‐2 region essential for transcriptional activation. Two phosphorylation sites are indicated: S51 in the DNA‐binding region and S208 within the hinge domain. Colored boxes represent functional regions as indicated in the legend: DNA‐binding (blue), nuclear localization motif (light green), heterodimerization (red), transcriptional activation (purple), and hormone‐binding domain (green). AF‐2, activation function‐2; RXR, retinoid X receptor; S208, serine 208; S51, serine 51; VDR, vitamin D receptor.

**FIGURE 2:**
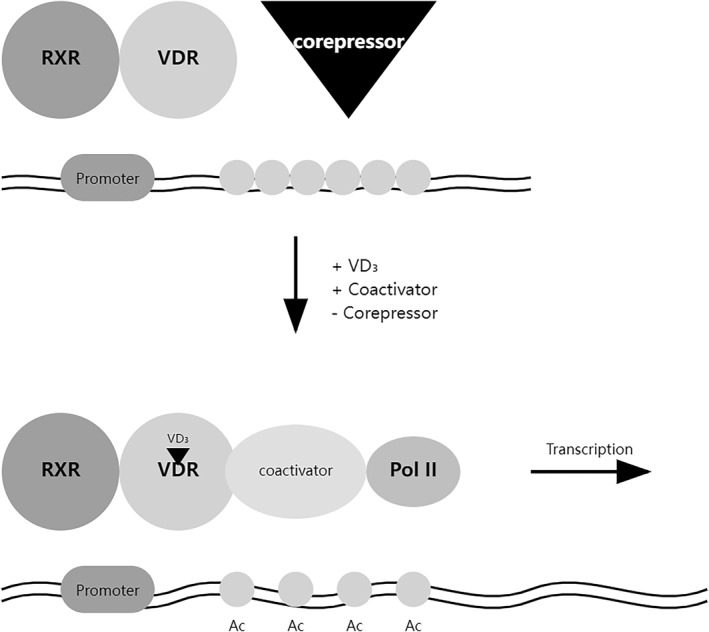
Schematic representation of VDR‐mediated transcriptional regulation. The upper panel shows the repressed state, where VDR–RXR heterodimer is associated with corepressor complexes at the promoter region. The lower panel illustrates the activated state following vitamin D (VD_3_) binding, where the corepressor is replaced by coactivator complexes. This exchange leads to histone Ac and recruitment of RNA Pol II, enabling transcriptional activation. The promoter region contains VDREs that serve as binding sites for the VDR–RXR heterodimer. This mechanism represents the canonical pathway for vitamin D‐dependent gene regulation. Ac, acetylation; Pol II, polymerase II; VDREs, vitamin D response elements; VDR–RXR, vitamin D receptor‐retinoid X receptor.

The VDR is a ligand‐dependent transcription factor belonging to the nuclear receptor superfamily, characterized by distinct functional domains including a highly conserved DNA‐binding domain (DBD) and a ligand‐binding domain (LBD).^22^ The DBD contains two zinc finger motifs that recognize specific vitamin D response elements (VDREs) in target gene promoters, while the LBD interacts with 1,25‐dihydroxyvitamin D3 (1,25(OH)2D3) and various coregulatory proteins^23^ (Figure 1). Upon ligand binding, VDR undergoes conformational changes that facilitate its heterodimerization with retinoid X receptor (RXR) and subsequent recruitment of coactivator complexes, leading to transcriptional activation of target genes^24^ (Figure 2). The transcriptional activity of VDR is further modulated by posttranslational modifications, including phosphorylation, ubiquitination, and SUMOylation, which fine‐tune its function in response to various cellular signals.^25^, ^26^, ^27^ In the skin, VDR plays fundamental roles in epidermal barrier formation by regulating keratinocyte differentiation and calcium homeostasis.^28^ It also contributes to cutaneous immune responses and inflammation through modulation of antimicrobial peptide production and T cell function.^29^ In hair follicles, VDR exerts both genomic and non‐genomic actions, regulating crucial biological processes such as proliferation, differentiation, and apoptosis.^30^ Notable examples of VDR‐regulated genes in hair follicles include those involved in keratin production, cell cycle regulation (p21 and cyclin D), and Wnt signaling, demonstrating its essential role in maintaining proper hair follicle homeostasis and cycling.^31^, ^32^, ^33^ Table 1 presents a curated summary of these genes, categorized by their functional roles and the experimental evidence supporting their regulation by VDR signaling. This intricate regulation of VDR signaling, through both structural modifications and diverse target gene networks, underscores its central role in maintaining skin barrier function and hair follicle homeostasis, making it a crucial therapeutic target for various dermatological conditions.^53^

**TABLE 1:** Summary table of VDR target genes in hair follicle biology.

Gene name	Functional category	Role in hair biology	Evidence type	Tissue/cell type	Reference(s)
CYP24A1	Vitamin D metabolism	Inactivation of 1,25(OH)_2_D_3_	Expression analysis	Hair follicle/skin	34, 35
CYP27B1	Vitamin D metabolism	Activation of vitamin D	Expression analysis	Hair follicle/skin	34, 35
DKK1	Wnt inhibitor	Canonical Wnt pathway inhibition	Transcriptional target	Hair follicle	36
SOSTDC1	Wnt/BMP inhibitor	Regulation of Wnt and BMP signaling	Transcriptional target	Hair follicle	36
KRTAPs	Structural proteins	Hair keratin‐associated proteins	Transcriptional target	Hair shaft/cortex	36, 37
Hair‐specific keratins	Structural proteins	Hair shaft formation	Transcriptional target	Hair follicle	36, 37
CDKN1A (p21)	Cell cycle regulation	Cell cycle arrest, differentiation	Transcriptional target	Keratinocytes/hair follicle	31, 38
Cyclin D	Cell cycle regulation	Cell proliferation control	Transcriptional target	Hair follicle matrix	31
Gli transcription factors	Hedgehog signaling	Hair follicle morphogenesis	Pathway interaction	Hair follicle	31, 39
Hairless	Hair cycle regulation	Catagen transition	VDR‐mediated control	Hair follicle	37, 40
Soggy	Unknown in hair	VDR‐mediated control	Transcriptional target	Keratinocytes	37
Wise	Wnt signaling modulator	VDR‐mediated control	Transcriptional target	Keratinocytes	37
Wnt pathway genes	Wnt signaling	Hair follicle differentiation	Pathway activation	Hair follicle/epidermis	31, 32, 41
Hedgehog pathway components	Hedgehog signaling	Hair follicle cycling	Pathway regulation	Hair follicle	31, 38, 39, 42, 43
Notch pathway components	Notch signaling	Stem cell maintenance	Pathway crosstalk	Hair follicle stem cells	44, 45
BMP pathway genes	BMP signaling	Hair cycle progression	Pathway modulation	Hair follicle	46
p63	Transcription factor	Epidermal cell fate and stem cell maintenance	Cross‐talk/co‐regulation	Epidermis/hair follicle	47
Antimicrobial peptides	Immune function	Cutaneous immune response	Transcriptional regulation	Epidermis/hair follicle	29
Lipid metabolism genes	Lipid metabolism	Sphingolipid production and barrier formation	ChIP‐seq identified	Epidermis	48, 49, 50
Cell adhesion molecules	Cell–cell interaction	Follicular structure maintenance	ChIP‐seq identified	Hair follicle	49, 50
ECM organization genes	Extracellular matrix	Follicular microenvironment	ChIP‐seq identified	Hair follicle/dermis	49, 50
Calcium homeostasis genes	Calcium regulation	Keratinocyte differentiation	VDR‐dependent regulation	Epidermis/hair follicle	28, 51
Differentiation markers	Keratinocyte differentiation	Epidermal barrier and hair formation	Transcriptional regulation	Epidermis/hair follicle	36, 52

Abbreviations: BMP, bone morphogenetic protein; ChIP‐seq, chromatin immunoprecipitation sequencing; ECM, extracellular matrix; VDR, vitamin D receptor.

### 2.2 VDR expression patterns in hair follicles

VDR exhibits dynamic expression patterns throughout the hair follicle structure, with particularly high levels in keratinocytes of the outer root sheath and matrix cells during late anagen and catagen phases.^54^ Immunohistochemical studies have revealed that VDR expression is not uniform across different hair follicle compartments, with distinct temporal and spatial patterns observed during various stages of the hair cycle.^55^ The receptor's expression is notably elevated in bulge stem cells and their immediate progeny, suggesting a crucial role in stem cell function and hair follicle renewal.^56^ During catagen, VDR expression shows a characteristic shift from the matrix to the regressing epithelial strand, indicating its involvement in the regulation of follicular regression and remodeling.^33^ VDR expression was reported to be regulated by a variety of hormones, including parathyroid hormone, retinoic acid, and the glucocorticoids.^57^, ^58^ Recent single‐cell RNA‐sequencing analyses have further revealed distinct VDR expression signatures in different hair follicle cell populations, providing new insights into its cell type‐specific functions and regulatory networks.^59^ VDR expression shows significant alterations in hair loss disorders, with clinical studies documenting markedly reduced VDR levels both in serum and follicular tissue of patients with AGA and AA compared to healthy controls.^60^ This VDR deficiency parallels impaired epidermal differentiation and reduced hair follicle growth,^52^ suggesting that VDR dysregulation is not merely associative but likely plays a causative role in disease pathogenesis, positioning VDR as a potential therapeutic target for hair loss.

### 2.3 Key molecular mechanisms of gene regulation and downstream targets

**FIGURE 3:**
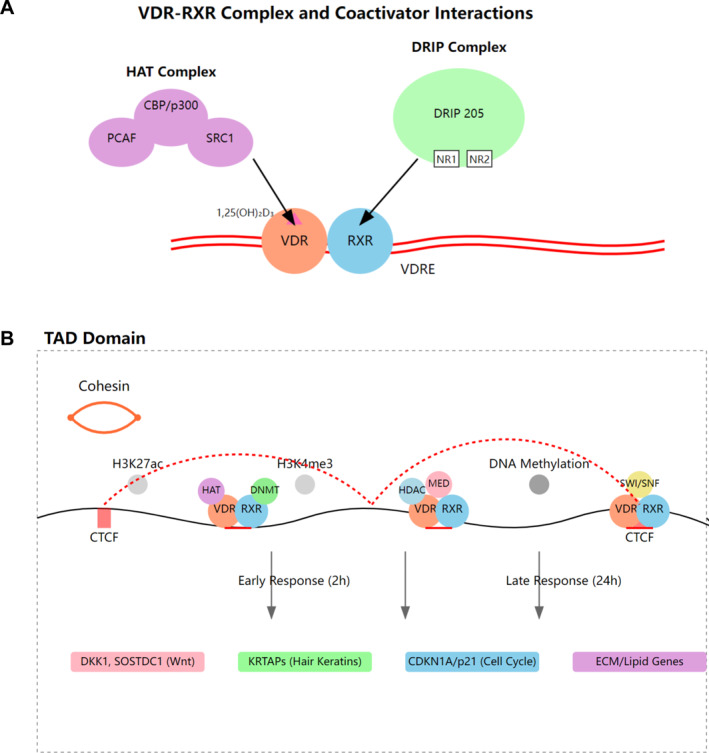
VDR–RXR‐mediated gene regulation from molecular complexes to chromatin organization. (A) Detailed structure of VDR–RXR heterodimer interactions with coactivator complexes. The HAT complex (comprising CBP/p300, PCAF, and SRC1) and the DRIP complex (containing DRIP205 with nuclear receptor binding motifs NR1 and NR2) associate with the VDR–RXR heterodimer bound to VDRE in the presence of 1,25(OH)_2_D_3_. (B) Chromatin architecture and sequential gene activation within a TAD domain. The diagram shows chromatin looping mediated by CTCF and cohesin, along with dynamic recruitment of different complexes (HAT, DNMT, HDAC, MED, and SWI/SNF) to VDR–RXR binding sites. Histone modifications (H3K27ac and H3K4me3) and DNA methylation status are indicated. Target genes are activated in a temporal sequence: early response genes (2 h) and late response genes (24 h). CTCF, CCCTC‐binding factor; DRIP, vitamin D receptor‐interacting protein; TAD, topologically associating domain; VDRE, vitamin D response element; VDR–RXR, vitamin D receptor‐retinoid X receptor.

The functionality of VDR in skin and hair follicles relies heavily on its interaction with essential molecular partners. The most crucial partner is RXR, which forms obligate heterodimers with VDR to enable efficient DNA binding and transcriptional regulation at VDRE.^17^ VDR also recruits various coactivators, including steroid receptor coactivators (SRCs) and the vitamin D receptor‐interacting proteins (DRIPs), which facilitate chromatin remodeling and transcriptional activation.^48^, ^61^, ^62^, ^63^ Mechanistically, upon ligand binding, VDR undergoes conformational changes that expose specific binding surfaces, allowing SRCs to interact with the activation function‐2 (AF‐2) domain while the DRIPs/MEDIATOR complex binds to both AF‐2 and other regions of VDR, collectively forming a multi‐protein complex that recruits histone acetyltransferases and other chromatin modifiers to create an open chromatin environment conducive to transcriptional activation^24^, ^64^, ^65^ (Figure 3A). Transcriptional regulation by VDR also involves complex spatial organization of chromatin, where VDR binds to enhancer regions and interacts with the basal transcriptional machinery at transcription start sites through DNA looping within defined topologically associating domains (TADs).^66^, ^67^ These TADs, which are large chromatin domains bordered by CCCTC‐binding factor (CTCF)‐binding sites and cohesin complexes, serve as functional regulatory units that constrain VDR‐mediated enhancer–promoter interactions, ensuring that VDR‐bound enhancers can only regulate genes within the same TAD and limit the maximum linear distance of regulatory interactions to the TAD size.^66^, ^67^, ^68^ VDR signaling has been reported to induce broad epigenetic reprogramming through multiple mechanisms, including modulation of active histone marks (H3K27ac and H3K4me3), DNA demethylation, widespread alterations in chromatin accessibility at enhancer and promoter regions, regulation of CTCF binding, and reorganization of three‐dimensional chromatin architecture through TAD modifications^69^, ^70^, ^71^, ^72^, ^73^ (Figure 3B). Of note, some VDR chromatin loci become accessible within 2 h of ligand stimulation while others require up to 24 h to reach maximal accessibility,^69^, ^74^ suggesting a temporal hierarchy in VDR‐mediated chromatin remodeling that involves both rapid direct effects and delayed secondary mechanisms.

Key downstream targets in hair follicles include the canonical Wnt inhibitors (DKK1 and SOSTDC1), hair keratin‐associated proteins, and hair‐specific keratins.^36^, ^37^ The receptor also regulates crucial cell cycle regulators such as CDKN1A (p21) and hedgehog signaling components, which are essential for proper hair follicle cycling and regeneration.^38^, ^42^, ^43^ Genome‐wide chromatin immunoprecipitation sequencing (ChIP‐seq) studies have revealed numerous novel VDR target genes involved in lipid metabolism, cellular adhesion, and extracellular matrix organization, expanding our understanding of regulatory network by VDR in hair biology.^49^, ^50^

### 2.4 Cross talk with other signaling pathways

VDR signaling exhibits extensive cross talk with other major regulatory pathways in hair follicles. A particularly important interaction exists with the Wnt/β‐catenin pathway, where VDR can either suppress or enhance Wnt signaling depending on the cellular context and presence of its ligand.^32^, ^41^ The receptor also interfaces with the Notch signaling pathway,^44^ which is crucial for hair follicle stem cell maintenance and fate decisions.^45^ VDR signaling modulates and is modulated by BMP signaling, creating a complex regulatory network that controls hair cycle progression.^46^ Additionally, there is significant cross talk between VDR and androgen receptor (AR) signaling pathways,^75^ though the functional relevance of this interaction in hair follicle development remains to be fully elucidated. The hedgehog signaling pathway, essential for hair follicle morphogenesis and cycling, also shows substantial cross talk with VDR, possibly through the regulation of Gli transcription factors.^31^, ^39^ Recent studies have also revealed important interactions with the mTOR signaling pathway, suggesting a role for VDR in metabolic regulation of hair follicle stem cells.^76^, ^77^, ^78^ This intricate network of pathway interactions highlights the complexity of VDR signaling and its central role in coordinating various aspects of hair follicle biology.

## 3 VDR IN HAIR FOLLICLE CYCLING

### 3.1 Role in anagen initiation

The VDR signaling plays a crucial role in initiating the anagen phase of the hair cycle. During this phase, VDR expression is markedly upregulated in the dermal papilla and hair matrix cells,^51^ where it coordinates the activation of hair follicle stem cells and subsequent proliferation of matrix keratinocytes.^79^ Through its interaction with key signaling pathways, particularly Wnt/β‐catenin^31^, ^32^ and Sonic hedgehog (Shh),^39^ VDR promotes the transition from telogen to anagen and supports the early stages of hair shaft formation.^80^

### 3.2 Regulation of catagen transition

VDR is intricately involved in regulating the catagen transition, a critical phase marked by programmed cell death and hair follicle regression.^81^ During this phase, VDR expression patterns shift significantly,^80^ and the receptor modulates the expression of pro‐apoptotic factors and cell cycle regulators.^51^ This carefully orchestrated process may function to ensure proper timing of catagen initiation and progression, with VDR acting as a key molecular switch in response to various regulatory signals.^40^

### 3.3 Impact on hair follicle stem cells

VDR exerts significant influence over hair follicle stem cell behavior and maintenance. The receptor is expressed in the bulge region where hair follicle stem cells reside,^56^ and its signaling is crucial for maintaining the stem cell pool^82^ and regulating their activation status.^83^ Through its interactions with various signaling pathways, VDR helps orchestrate the delicate balance between stem cell quiescence and activation in hair follicle stem cells,^21^, ^56^, ^84^, ^85^ which is essential for sustainable hair follicle cycling and regeneration. This regulatory role of VDR in stem cell maintenance appears to be a conserved mechanism across different tissue types, as demonstrated by its critical function in controlling stemness in hematopoietic cells^86^ and colon stem cells.^87^ Recent studies have revealed that VDR achieves this regulation through cross talk with other key transcription factors, particularly p63, which promotes epidermal cell fate determination and stem cell maintenance.^47^ This complex interplay between VDR and various signaling pathways underscores its fundamental role in stem cell homeostasis and tissue regeneration.

## 4 CLINICAL EVIDENCE OF VDR DYSFUNCTION IN HAIR LOSS DISORDERS

### 4.1 Lessons from VDR knockout models and VDR polymorphisms

Studies using VDR knockout mice have provided crucial insights into the role of VDR in hair biology. These models demonstrate progressive alopecia starting after the first hair cycle, indicating VDR's essential role in hair follicle cycling.^18^, ^39^, ^88^ Various VDR polymorphisms and mutations have been identified in human populations, with some variants showing strong associations with different types of hair loss.^54^, ^89^ Notably, specific VDR mutations affect its DNA‐binding capacity and transcriptional activity, which may lead to aberrant regulation of genes involved in hair follicle cycling and maintenance.^90^, ^91^ The phenotypic similarities between VDRKO mice and humans with HVDRR who harbor VDR mutations further support the critical role of VDR signaling in hair follicle homeostasis.^18^, ^92^, ^93^

### 4.2 Vitamin D deficiency and alopecia

While VDR functionality is crucial for hair follicle maintenance, the relationship between vitamin D deficiency and hair loss presents a complex picture. Clinical studies have shown that vitamin D deficiency is prevalent among patients with various forms of alopecia.^94^, ^95^, ^96^ However, the causative relationship remains debatable, as some VDR functions in hair follicles appear to be ligand‐independent.^81^, ^97^, ^98^ This distinction is critically illustrated by comparing VDR‐null mice, which develop complete alopecia after the first hair cycle, with CYP27B1‐null mice, which lack the enzyme to produce active vitamin D but maintain functional VDR and exhibit normal hair cycling.^81^, ^88^ This divergence demonstrates that VDR protein presence, rather than ligand binding, is the primary determinant of hair follicle maintenance. Nevertheless, severe vitamin D deficiency has been associated with telogen effluvium and diffuse hair loss, suggesting that optimal vitamin D levels are necessary for maintaining healthy hair growth cycles.^4^ Supplementation studies have shown varying degrees of success in treating vitamin D deficiency‐related hair loss, indicating the importance of maintaining adequate vitamin D status.^99^, ^100^

### 4.3 Role in specific types of alopecia: AA, AGA, and telogen effluvium

Different types of alopecia demonstrate distinct relationships with VDR function and vitamin D status. In AA, an autoimmune condition is exhibited, while VDR gene polymorphism shows no direct association,^101^, ^102^ patients exhibit reduced VDR expression,^60^, ^103^ and vitamin D supplementation has demonstrated promising therapeutic outcomes in some cases.^104^ AGA presents a complex interplay between VDR signaling and androgen pathways, with studies indicating that VDR polymorphisms may influence disease susceptibility.^105^ Furthermore, patients with AGA show both reduced VDR expression and a higher prevalence of low serum vitamin D levels.^60^, ^94^ In telogen effluvium, vitamin D deficiency appears to function as both a potential trigger and a maintaining factor, with evidence suggesting that normalizing vitamin D levels can aid recovery.^106^ A significant case report revealed that specific VDR gene polymorphisms (Taq1 CC genotype and Cdx1 GA genotype) were markedly more prevalent in chronic telogen effluvium patients than controls, with the CC genotype increasing disease risk by 14.7‐fold and the GA genotype by 6.3‐fold, suggesting these genetic variations may contribute to persistent hair loss by inhibiting new hair growth and stem cell proliferation.^107^ These findings collectively underscore the critical role of VDR signaling and vitamin D homeostasis in various forms of alopecia, suggesting that therapeutic strategies targeting this pathway may offer promising treatment options for different types of hair loss disorders.

## 5 THERAPEUTIC APPLICATIONS AND PERSPECTIVES IN HAIR LOSS PATIENTS

### 5.1 Vitamin D status assessment in hair loss patients

Accurate assessment of vitamin D status has become increasingly important in the management of hair loss disorders. Current guidelines recommend measuring serum 25‐hydroxyvitamin D levels as the primary biomarker, with values below 20 ng/mL indicating deficiency and 21–29 ng/mL suggesting insufficiency.^108^ Regular monitoring is particularly crucial in patients with chronic hair loss conditions, as studies have shown a high prevalence of vitamin D deficiency in this population.^109^ However, emerging research suggests that local vitamin D metabolism in the hair follicle might not always correlate with systemic levels,^110^ necessitating the development of several tissue‐specific assessment methods. These include direct measurement of metabolite levels in skin tissue samples using liquid chromatography‐mass spectrometry (LC‐MS/MS)^111^, ^112^ and assessment of local CYP27B1 and CYP24A1 enzyme expression.^34^, ^35^ Noninvasive techniques could also applied, such as tape stripping methods combined with LC‐MS/MS analysis to measure metabolites in the stratum corneum^113^ and microdialysis techniques to assess dermal interstitial fluid.^114^ Additionally, molecular markers including vitamin D‐dependent gene expression profiles in scalp tissue, local inflammatory markers associated with vitamin D deficiency, and keratinocyte differentiation markers regulated by vitamin D provide further insights into tissue‐specific vitamin D status.^67^ These advanced assessment methods, when integrated with conventional serum measurements, offer a more comprehensive understanding of vitamin D homeostasis in hair loss disorders and may help optimize treatment strategies.

### 5.2 Current therapeutic approaches: Vitamin D supplementation, topical vitamin D analogs, and combination therapies

Treatment strategies involving vitamin D have evolved significantly, with approaches tailored to specific types of alopecia. Oral supplementation protocols typically recommend doses ranging from 1000 to 4000 IU daily, with higher doses considered for severe deficiency states.^100^, ^115^ Topical vitamin D analogs, such as calcipotriol and calcitriol, have shown promise particularly in treating AA, with studies reporting improved hair regrowth when combined with corticosteroids.^104^, ^116^, ^117^, ^118^, ^119^ Combination therapies have emerged as particularly effective approaches, integrating vitamin D supplementation with conventional treatments. For instance, the combination of vitamin D with minoxidil has demonstrated enhanced efficacy in female pattern hair loss and chemotherapy‐induced alopecia.^99^, ^120^ Given the immunomodulatory role of vitamin D, its supplementation is expected to show synergistic effects with other immunotherapies in treating AA, an autoimmune condition, through complementary mechanisms of immune response modification and promotion of hair follicle regeneration.^95^, ^121^

### 5.3 Novel therapeutic perspectives: Emerging VDR‐targeted therapies and drug delivery systems

The future of alopecia treatment is being shaped by innovative approaches targeting VDR signaling and improved delivery systems. Novel VDR agonists and vitamin D derivatives have demonstrated promising therapeutic potential through both VDR‐dependent and VDR‐independent mechanisms, with recent studies showing that compounds like EB1089 can exert biological effects independently of VDR,^122^ while new structural modifications of vitamin D analogs can enhance their interaction with dysfunctional VDR variants,^123^ providing multiple therapeutic avenues that can be evaluated through advanced in vitro and in vivo screening systems.^124^, ^125^ While various VDR ligands have demonstrated enhanced therapeutic efficacy in experimental models of cancer, inflammation, and cardiovascular disease with evidence of function‐selective and tissue‐selective activities,^126^ their potential therapeutic applications and underlying molecular mechanisms in different forms of alopecia remain largely unexplored and warrant further investigation.

Advanced drug delivery systems, including nanocarriers and follicle‐targeted formulations, are being developed to enhance the efficacy of vitamin D‐based treatments.^127^, ^128^ Liposomal preparations and novel penetration enhancers have shown improved follicular delivery of vitamin D derivatives and topical vitamin D analogs.^129^, ^130^ The optimization of these delivery systems involves extensive in silico screening of chemical permeation enhancers using molecular dynamics simulations to predict and enhance skin permeability.^131^ Recent ex vivo studies have also investigated the feasibility of transdermal vitamin D3 formulations, focusing on skin retention and permeation characteristics to improve therapeutic efficacy,^132^ highlighting the ongoing efforts to develop more effective topical delivery strategies for skin disorders. In the context of vitamin D delivery through the skin, niosomeness show potential advantages due to their nonionic surfactant composition, which may enhance vitamin D stability and reduce irritation,^133^ while nanocrystals could improve the bioavailability of poorly water‐soluble vitamin D analogs.^134^ Natural lipid‐based nanoparticles, particularly those incorporating oils with inherent skin benefits,^135^, ^136^ represent a promising approach for co‐delivery of vitamin D with other bioactive compounds. VDR variants and dysregulation are widely observed in HVDRR, leading to alopecia and variable clinical responses.^137^ Gene therapy approaches, including targeted expression of VDR in keratinocytes^138^ and the use of VDR‐expressing adenovirus vectors,^139^ have demonstrated promising results in preventing and treating alopecia in experimental models. Combined with advanced drug delivery systems, including nanocarriers and tissue‐specific targeting strategies, these therapeutic approaches may provide novel treatment options for patients with VDR‐related hair disorders.

## 6 CHALLENGES AND FUTURE DIRECTIONS

### 6.1 Current limitations in understanding

While significant progress has been made in understanding VDR signaling in hair biology, several key knowledge gaps remain. The precise mechanisms by which VDR coordinates with other nuclear receptors in hair follicle regulation are not fully understood.^140^ Further, since VDR signaling mediates a myriad of biological actions,^141^ understanding the temporal and spatial specificity of VDR actions during different hair cycle phases could help develop more targeted therapies that minimize off‐target effects while maximizing therapeutic efficacy in the hair follicle. Additionally, the relationship between systemic vitamin D levels and local VDR signaling in hair follicles remains poorly defined, particularly regarding how circulating vitamin D metabolites influence VDR‐mediated transcriptional activity in different hair follicle cell populations and how this relationship might be altered in various pathological conditions.

While VDR polymorphisms have been identified in patients with alopecia, their functional implications for hair growth and treatment outcomes remain poorly understood, making it challenging to predict individual responses to vitamin D‐based therapies. Elucidating the mechanistic defects and responses associated with each VDR variant could facilitate the development of personalized therapeutic strategies tailored to individual genetic profiles, thereby potentially enhancing treatment efficacy. The complexity of VDR's dual functions (ligand‐dependent and ligand‐independent, Table 2) in hair follicle regulation presents challenges for therapeutic development, particularly concerning its interaction with inflammatory mediators and its role in immune system development and function.^142^, ^143^ The stark phenotypic difference between VDR‐null mice (which develop alopecia) and CYP27B1‐null mice (which maintain normal hair despite lacking active vitamin D) underscores that many critical VDR actions in hair follicles operate independently of ligand binding.^81^, ^88^, ^98^ Consequently, there is a pressing need to decipher how VDR dysfunction influences the local immune microenvironment and its subsequent impact on follicle cycling, as this knowledge could reveal novel therapeutic targets and approaches for treating various forms of alopecia.

**TABLE 2:** Ligand‐dependent versus ligand‐independent action of VDR.

Feature	Ligand‐dependent	Ligand‐independent
Requires 1,25(OH)_2_D_3_	✓ Yes	✗ No
Partner protein	RXR (obligate heterodimer)	β‐catenin, p63, and other transcription factors
DNA‐binding site	VDRE (DR3 elements)	Non‐VDRE and context‐dependent
Coregulators	SRC, p300/CBP, and DRIP/mediator	Distinct complexes, corepressors
Hair follicle development	Important (++)	Essential (+++)
Stem cell maintenance	Moderate (+)	Critical (+++)
Primary functions	Gene transcription, differentiation	Development and stem cell regulation
Evidence model	VDR + ligand studies	CYP27B1‐null versus VDR‐null mice

Abbreviations: DRIP, vitamin D receptor‐interacting protein; RXR, retinoid X receptor; VDR, vitamin D receptor; VDRE, vitamin D response element.

Another significant knowledge gap concerns sex‐specific differences in VDR signaling within hair follicles. While AGA exhibits clear sexual dimorphism in presentation and progression,^10^, ^11^ and VDR interacts with AR signaling pathways,^75^ there is a notable lack of studies investigating whether VDR expression, activity, or downstream target gene regulation differs between male and female hair follicles. Given that sex hormones can modulate VDR expression in other tissues^57^, ^58^ and that treatment responses to vitamin D‐based therapies may vary between sexes, understanding potential sex‐specific mechanisms of VDR action in hair biology could be crucial for developing personalized therapeutic approaches.

The role of VDR in hair follicle aging and its interaction with environmental factors needs further investigation,^144^, ^145^ especially regarding age‐related hair loss and the impact of conditions like oxidative stress on VDR function. Understanding how aging‐associated changes in VDR expression and activity contribute to hair follicle dysfunction could provide new therapeutic strategies for age‐related hair disorders. Recent evidence has demonstrated that various metabolic disorders can significantly alter VDR expression patterns in multiple tissues.^146^, ^147^ Understanding how metabolic disorders and systemic diseases affect VDR expression and signaling in hair follicles remains crucial for developing more effective treatments for patients with complex medical conditions. This knowledge could help identify novel therapeutic targets and develop combination treatments that address both the underlying systemic condition and its impact on hair follicle health through VDR‐mediated pathways.

### 6.2 Technical challenges and research gaps

Current technical limitations include several significant challenges in VDR research and therapeutic development. The real‐time monitoring of VDR activity in hair follicles remains difficult due to the limitations of current imaging techniques and the complex three‐dimensional structure of hair follicles. While fluorescent reporters exist, they often fail to capture the dynamic nature of VDR signaling in living tissue.^148^ The lack of reliable models that fully replicate human hair disorders presents another major obstacle. Although various animal models and in vitro systems exist, they often fail to accurately represent the complexity of human hair follicle biology and disease states. Mouse models, while valuable, show significant differences in hair cycling patterns and immune responses compared to humans.^149^ The complexity of isolating and maintaining primary hair follicle cells while preserving their natural characteristics poses significant challenges for in vitro studies.^150^ Current culture systems often result in the loss of crucial cell–cell interactions and the natural microenvironment that supports proper follicular function. This limitation particularly affects studies of dermal papilla cells, which rapidly lose their hair‐inductive properties when cultured in conventional systems.^151^ Another major challenge lies in the development of tissue‐specific delivery systems. Creating formulations that can effectively target hair follicles while minimizing systemic exposure remains difficult. The complex architecture of the hair follicle, variations in follicular penetration, and the challenge of maintaining therapeutic concentrations at the target site all contribute to this difficulty.^152^ Furthermore, the blood–follicle barrier presents additional obstacles for drug delivery, particularly for larger molecules and biologics designed to target VDR signaling pathways.^153^

### 6.3 Future research priorities

**FIGURE 4:**
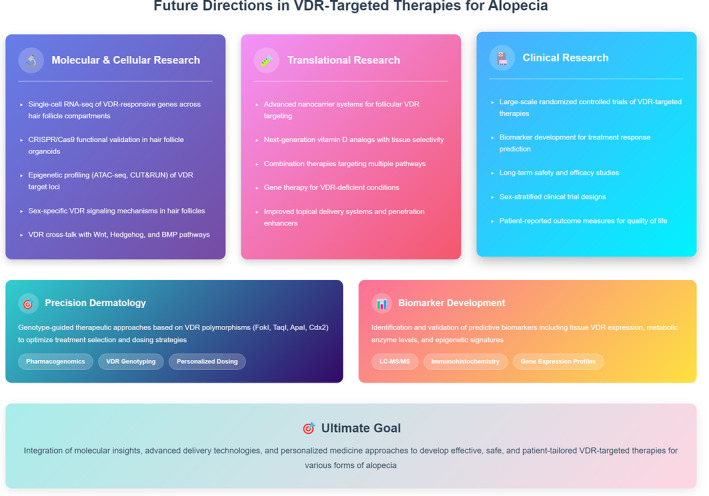
Future research priorities for VDR‐targeted therapies in alopecia. The diagram outlines key research areas including molecular and cellular investigations (single‐cell technologies, CRISPR/Cas9 validation, and epigenetic profiling), translational research (nanocarrier development, next‐generation analogs, and combination therapies), and clinical research (biomarker discovery and randomized controlled trials). Integration of precision dermatology approaches based on VDR polymorphisms and validated biomarkers will enable personalized therapeutic strategies for effective treatment of hair loss disorders. VDR, vitamin D receptor.

Future research priorities should focus on several interconnected aspects of VDR biology at the molecular and cellular levels. The foundation lies in developing improved methods for studying VDR signaling in human hair follicles, which will enable more accurate investigation of VDR's role in different hair follicle cell populations through single‐cell technologies.^154^ Advanced single‐cell RNA‐sequencing approaches could provide unprecedented resolution in identifying VDR‐responsive genes across distinct hair follicle compartments and developmental stages, revealing cell type‐specific transcriptional programs that remain poorly characterized. Additionally, the application of CRISPR/Cas9 technology to hair follicle organoid models and in vivo systems would enable precise functional validation of VDR signaling mechanisms and could accelerate the identification of therapeutic targets. This enhanced understanding should be complemented by studies on epigenetic regulation of VDR signaling in hair cycle control, as epigenetic modifications may significantly influence treatment responses.^155^ Future epigenetic profiling studies, particularly utilizing techniques such as ATAC‐seq and CUT&RUN in hair follicle stem cells, could elucidate how chromatin accessibility at VDR target loci is dynamically regulated during hair cycling and how these patterns differ in various forms of alopecia. Systematic investigation of VDR cross talk with different signaling pathways could further reveal synergistic mechanisms and inform our understanding of how VDR integrates multiple developmental signals in the hair follicle niche (Figure 4).

Building on these molecular insights, translational research should prioritize the development of more effective therapeutic strategies. Efforts should be directed toward creating more efficient drug delivery systems specifically targeting VDR pathways in hair follicles, which remains a significant technical challenge. Development of next‐generation vitamin D analogs with enhanced tissue selectivity and reduced systemic effects represents another promising avenue, potentially overcoming limitations of current topical formulations such as calcipotriol and maxacalcitol. As our understanding of pathway cross talk deepens, exploring combination therapies that target multiple pathways alongside VDR signaling becomes crucial, potentially leading to more effective treatments. This approach should be coupled with efforts to establish personalized treatment strategies based on VDR genetic polymorphisms, as these variants significantly influence VDR mRNA expression, vitamin D metabolism, and treatment responsiveness across various conditions.^156^, ^157^, ^158^ This genetic understanding, coupled with comprehensive clinical evaluations, could enable the development of genotype‐guided therapeutic approaches to optimize treatment efficacy in hair disorders. To ensure successful clinical implementation, rigorous evaluation of novel VDR‐targeted therapeutics through well‐designed clinical trials is essential, with particular focus on long‐term safety profiles and treatment durability. Furthermore, the identification and validation of reliable biomarkers for predicting treatment response in different types of alopecia will be crucial for enabling more precise, patient‐tailored therapeutic approaches and optimizing clinical outcomes (Figure 4).

## 7 CONCLUSION AND CLINICAL IMPLICATIONS

The evolving understanding of VDR signaling in hair biology has profound implications for both research and clinical practice. The complex interplay between VDR and various signaling pathways, including Wnt/β‐catenin, Notch, and hedgehog signaling, underscores its central role in hair follicle homeostasis and cycling. Recent advances in understanding VDR genetic polymorphisms^156^, ^157^, ^158^ and their impact on treatment responses have opened new avenues for personalized therapeutic approaches. While conventional treatments such as vitamin D supplementation and topical analogs remain valuable, emerging strategies including advanced drug delivery systems, targeted therapies, and combination treatments offer promising alternatives for managing various forms of alopecia. The development of tissue‐specific delivery systems and biomarkers for predicting treatment response will be crucial for optimizing therapeutic outcomes. Furthermore, the recognition of VDR's role in hair follicle stem cell maintenance and immune regulation suggests potential applications in regenerative medicine and autoimmune hair disorders. As research continues to unravel the complexities of VDR signaling, the integration of molecular insights with clinical applications will be essential for developing more effective, personalized treatments for patients with hair disorders. This progress, coupled with advances in diagnostic technologies and therapeutic delivery systems, positions VDR‐targeted approaches as a cornerstone in the future management of alopecia and related conditions.

## AUTHOR CONTRIBUTIONS

The authors confirm contribution to the paper as follows: study conception and design: Liancheng Guan and Yunzhi Chen; draft manuscript preparation: Liancheng Guan and Fan Yang; Literature collation: Meijuan Li, Yujia Chen and Zexin Zhao; Framework: Hongxia Li, Deping Luo and Qian Li. All authors reviewed the results and approved the final version of the manuscript.

## CONFLICT OF INTEREST STATEMENT

The authors declare no conflicts of interest.

## ETHICS STATEMENT

Not applicable.

## CLINICAL TRIAL NUMBER

Not applicable.

## Data Availability

The data generated in this study are available upon request to the corresponding author.
